# Supplementation of Maternal Diets with Docosahexaenoic Acid and Methylating Vitamins Impacts Growth and Development of Fetuses from Malnourished Gilts

**DOI:** 10.3945/cdn.117.001958

**Published:** 2017-12-08

**Authors:** Hope K Lima, Xi Lin, Sheila K Jacobi, Caolai Man, Jeffrey Sommer, William Flowers, Anthony Blikslager, Liara Gonzalez, Jack Odle

**Affiliations:** 1 Laboratory of Developmental Nutrition, North Carolina State University, Raleigh, NC; 2 Departments of Animal Science, North Carolina State University, Raleigh, NC; 3 Departments of Clinical Sciences, North Carolina State University, Raleigh, NC

**Keywords:** choline, B-vitamins, epigenetics, intrauterine growth restriction, nutrient restriction, low birth weight

## Abstract

**Background:**

Like many species, pregnant swine mobilize and repartition body nutrient stores during extreme malnutrition to support fetal development.

**Objective:**

The objective of this study was to model chronic human maternal malnutrition and measure effects of methylating-vitamins (MVs, containing choline, folate, B-6, B-12, and riboflavin) and docosahexaenoic acid (DHA) supplementation on fetal growth and development.

**Methods:**

Pregnant gilts (*n* = 24) were either fully nourished (2.0 kg/d) with a corn-plus-isolated-soy-protein basal diet (control) supplemented with MVs and DHA or nourishment was restricted throughout gestation. Basal diet fed to malnourished gilts was reduced progressively from 50% to 70% restriction (1.0 to 0.6 kg/d) and was supplemented following a 2 (±MVs) x 2 (±DHA) factorial design. Full-term c-sections were performed to assess impacts on low and normal birth weight (LBW/NBW) fetuses (*n* = 238).

**Results:**

Body weight gain of malnourished gilts was 10% of full-fed control dams (*P* < 0.05), but offspring birth weight, length, girth, and percentage of LBW fetuses were not different between treatments. The number of pigs per litter was reduced by 30% in malnourished control dams. Fetal brain weights were reduced by 7% compared to positive controls (*P* < 0.05). Micronutrient supplementation to malnourished dams increased fetal brain weights back to full-fed control levels. Dams with DHA produced offspring with higher DHA concentrations in brain and liver (*P* < 0.05). Plasma choline concentration was 4-fold higher in fetuses from unsupplemented malnourished dams (*P* < 0.0001). Global DNA methylation status of fetuses from restricted dams was higher than in control fetuses, including brain, liver, heart, muscle, and placenta tissues (*P* < 0.05). Addition of DHA increased methylation in LBW fetal brains (*P* < 0.05).

**Conclusions:**

Despite the mobilization of maternal stores, malnourished litters displayed reduced brain development that was fully mitigated by micronutrient supplementation. Severe maternal malnutrition increased global DNA methylation in several fetal tissues that was unaltered by choline and B-vitamin supplementation.

## Introduction

Micronutrient monitoring and supplementation during pregnancy in first-world countries is standard practice (1, 2), but developing nations struggle with dietary variability for provision of adequate micronutrients (3). Chronic undernutrition in the mother influences the availability of nutrients for fetal growth and development. Undernutrition is a global health issue, which can lead to neural tube defects, iron-deficiency anemia, insulin resistance, and cardiac dysfunction (4). Malnourishment during pregnancy is also associated with increased rates of intrauterine growth

restriction (IUGR), and low birth weight (LBW) is a leading factor contributing to infant morbidity and mortality worldwide (5).

Minor nutritional modifications during pregnancy may be able to favorably alter the metabolic phenotype of the offspring, decreasing the chances of IUGR, neonatal mortality, and chronic illness as an adult (6, 7). Additionally, nutritional changes may cause long-term metabolic effects by influencing metabolic programming of the fetus (8, 9). Several review articles offer extensive overviews linking nutritional status in utero to epigenetic changes, metabolic programming, and chronic adult disease (6, 7, 10). Despite this emerging link between optimal nutritional intake and epigenetic modification, the effect of the simultaneous supplementation of vitamins supporting one-carbon metabolism (choline, folate, B-6, B-12, and riboflavin) during nutrient deprivation on physiology and metabolism of the placenta and the fetus is not well characterized.

One-carbon metabolism utilizes key micronutrients as cofactors, intermediaries, and methyl donors to provide methyl groups for DNA methylation (11), which serves as one form of epigenetic regulation. Interestingly, DHA interacts with choline, a key methyl donor, as phosphatidylcholine DHA. Through this interaction, DHA supplementation has been linked to changes in one-carbon metabolism (8, 12, 13), and reduced DHA concentrations may divert methyl groups through the one-carbon metabolic pathway to increase DNA methylation (13). Conversely, increased concentrations of DHA may serve as a regulatory factor in choline metabolism (8).

When designing a targeted nutritional intervention option for use during pregnancy in situations of nutrient limitation or deprivation, substrates of one-carbon metabolism should be of interest. Specifically, we hypothesized that adequate supplementation of riboflavin (B-2), pyridoxine (B-6), folate (B-9), cobalamin (B-12), choline, and DHA may cause distinguishable physiologic and metabolic changes in the developing fetus when the mother is experiencing chronic undernutrition.

Swine are litter bearing (14, 15), and so nutrients delivered to the mother are distributed differently to the fetuses depending on their location along the uterine horn. This causes littermates to develop unique physiologic phenotypes, allowing for assessment of differences influenced specifically by nutritional availability, while minimizing differences influenced by genetics. Here, we studied LBW and normal birth weight (NBW) littermates to determine the effect of malnourishment on fetal growth and development. Additionally, the collective role that riboflavin (B-2), pyridoxine (B-6), folate (B-9), cobalamin (B-12), choline, and DHA supplementation play in fetal growth was investigated. By comparing data from LBW and NBW, we aimed to assess impacts that could lead to greater understanding of the effect of chronic maternal undernutrition. Additionally, the use of this combination of nutrients as an interventional method was assessed.

## Methods

### Experimental timeline

**FIGURE 1:**
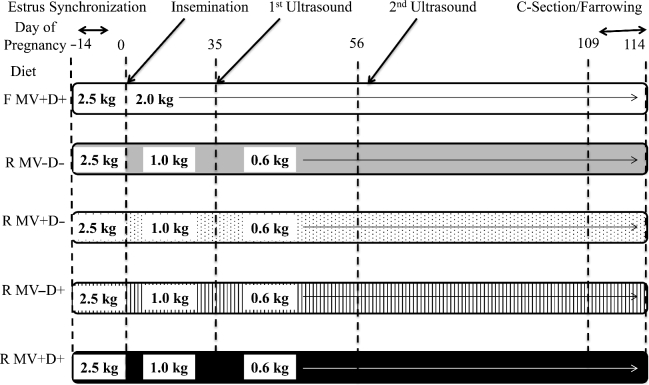
Timeline of reproductive events and nutritional treatments. Numbers contained within the boxes are representative of the amount of feed delivered (in kilograms). Groups are *n* = 5, *n* = 6, *n* = 4, *n* = 5, and *n* = 4, respectively. F MV+D+, positive control, full-fed supplemented with methylating vitamins and DHA; R MV–D–, negative control, restricted basal feed without methylating vitamins or DHA; R MV+D–, restricted basal feed supplemented with methylating vitamins only; R MV–D+, restricted basal feed supplemented with DHA only; R MV+D+, restricted basal feed supplemented with methylating vitamins and DHA.

Twenty-four 6–8-mo-old gilts (Landrace x Yorkshire x Duroc) were housed individually at the North Carolina State University Swine Educational Unit in Raleigh, North Carolina and fed 1 time/d. The gilts (303.5 ± 8.2 kg) then were randomly assigned to treatment groups (**Figure 1**). All protocols were approved by the North Carolina State University Animal Care and Use Committee.

Positive control gilts (*n* = 5) received a corn-plus-isolated-soy-protein diet (**Table 1**) containing 3322 kcal/kg metabolic energy, 13.2%

crude protein with 0.6% lysine, 0.2% Met, 0.39% Met + Cys, 0.85% calcium, and 0.63% phosphorus and met all nutrient requirements (14). Isolated soy protein was used instead of soybean meal in order to minimize the choline content of the basal diet. The diet was further supplemented with a mixture of methylating vitamins (MVs) containing folic acid (1.3 mg/kg feed), pyridoxine (1.0 mg/kg feed), B-12 (0.015 mg/kg feed), riboflavin (3.75 mg/kg feed), choline (1250 mg/kg feed, vitamins donated by DSM, Heerlen, Netherlands), and DHA (2420 mg/kg feed; life's DHA S35-O200, rosemary free algal vegetable oil, minimum 35% DHA; DSM, Columbia, MD; **Table 2**). Basal diet allotment to restricted gilts (*n* = 4–6/treatment) was supplemented according to a 2 (±MVs) x 2 (±DHA) factorial design (Table 2 and Figure 1). Restricted gilts were supplemented with the MV premix and DHA at the same rate as the control gilts (Figure 1).

**TABLE 1:** Basal diet composition

Ingredients	g/100 g of feed
Corn, yellow dent	89.34
Soy protein isolate^1^	7.26
Dicalcium phosphate 18.5%	1.84
Limestone	0.95
Salt	0.50
Basal vitamin premix^2^	0.06
Trace mineral premix^3^	0.05
Total	100.00

1 From Archer Daniels Midland, Chicago, IL.

2 Basal vitamin premix provided vitamin A, 17.64 g/kg premix; vitamin D, 7.06 g/kg premix; vitamin E, 388.01 g/kg premix; menadione, 6.68 g/kg premix; biotin, 8.82 g/kg premix; niacin, 44.09 g/kg premix; pantothenic acid, 58.79 g/kg premix; thiamin, 4.79 g/kg premix.

3 Trace mineral premix included manganese sulfate 6.00%, zinc sulfate 6.00%, ferrous sulfate 4.00%, copper sulfate 0.5%, calcium iodate 0.125%, cobalt sulfate 0.05%, and calcium carbonate as carrier.

**TABLE 2:** Experimental MVs and DHA expected supplementation rates^1^

Nutrients	F MV+D+	R MV–D–	R MV+D–	R MV–D+	R MV+D+
MVs					
Folic acid	2.6	0	2.6	0	2.6
Pyridoxine	2.0	0	2.0	0	2.0
B-12	0.030	0	0.030	0	0.030
Riboflavin	7.5	0	7.5	0	7.5
Choline	2500	0	2500	0	2500
Fatty acids					
DHA	4840	0	0	4840	4840

1 Expected supplementation rates expressed in mg/d. Other vitamins were contained in the basal diet (Table 1). F MV+D+, positive control, full-fed supplemented with methylating vitamins and DHA; MV, methylating vitamin; R MV–D–, negative control, restricted basal feed without methylating vitamins or DHA; R MV+D–, restricted basal feed supplemented with methylating vitamins only; R MV–D+, restricted basal feed supplemented with DHA only; R MV+D+, restricted basal feed supplemented with methylating vitamins and DHA.

Basal nutrients were delivered at a standard rate as determined by the NRC guide for gestation sows (14). Feeding rates were determined with reference to standard gestation feeding rates for sows (16, 17). Micronutrients (MVs and DHA) were pre-weighed, packaged, and stored in the dark at 4°C. Oil-based DHA was stored in an airtight container at 4°C. Both MVs and DHA were added to the basal diet allotments for each gilt immediately prior to feeding each day.

Gilts began receiving assigned diets 2 wk prior to breeding at a rate of 2.5 kg feed/d. Upon insemination, positive control gilts were fed 2.0 kg feed/d for the remainder of the trial. Restricted gilts were reduced to 1.0 kg basal diet/d (50% feed restriction), and the MVs and the DHA were fed according to their respective treatment assignment (Figure 1; Table 2).

Ultrasounds were performed on each gilt 35 and 56 d after insemination. When confirmed pregnant on day 35, the feed allotment to restricted gilts was further reduced to 0.6 kg/d (70% feed restriction) for the remainder of the trial.

### Synchronization and insemination

Fifteen milligrams of Matrix (Altrenogest; Intervet, Millsboro, DE) was delivered with feed daily for 14 d to synchronize estrus (Figure 1). Gilts were bred when in full standing heat for a maximum of 3 consecutive days. All gilts were inseminated using semen from the same sire to minimize genetic differences between fetuses. Semen was collected 1 time/wk and extended with USA851 X-Cell Extender (18). Semen was used within 5 d of collection.

### Caesarian sections and sample collection

In preparation, gilts were given an initial dose of anesthetics consisting of a 50/50 mixture of ketamine and xylazine at a dosage of 2.2 mg/kg body weight. This sedative was administered into a marginal ear vein. A surgical level of anesthesia was achieved using a closed-circuit anesthesia machine, which delivered 1.0% isoflurane in a mixture of oxygen and nitrous oxide.

The uterus was exposed via a 40-cm midventral incision. Blunt dissection was used to separate adipose tissue from the underlying connective tissue layers and expose the linea alba. A small puncture was made in the linea alba and the abdominal cavity was opened by cutting along the linea alba.

The uterine horns were manually removed from the abdominal cavity and a blood sample was taken from a branch of the uterine artery and collected in EDTA anticoagulant vacutainers. Beginning at the end adjacent to an ovary, fetuses were removed individually by making incisions along the longitudinal axis of the uterine horn. A 2.5-cm section of the fetal portion of the placenta was removed, frozen in liquid nitrogen, and stored at –80°C. All fetuses were then removed from the amniotic sac and blood samples were obtained via cardiac puncture.

Fetuses immediately were subjected to total exsanguination and their wet weight, sex, crown-to-rump length, and heart girth were recorded. Immediately following, each piglet was dissected, liver, heart, and brain (frontal cortex) were collected and weighed, and a skeletal muscle sample was taken from the right, posterior biceps femoris. All samples were frozen in liquid nitrogen, and stored at –80°C. This procedure was repeated for each piglet in the litter. Whole blood samples were centrifuged at 2000 × *g* for 15 min at 4°C and the plasma was collected and stored at –80°C.

### Sample analysis

#### GC-MS

The concentrations of DHA and other fatty acids in liver and brain tissues were analyzed using GC-MS. Fatty acid methyl esters were prepared from the tissue samples following the direct methylation method described by Wang et al. (19). The fatty acid methyl esters then were separated on an HP-23 capillary column (*cis/trans* FAME CR), 30 m × 0.25 mm, film thickness 0.3 µm (Agilent Technologies, Wilmington, DE). Mass spectrometric analysis was conducted by using an Agilent Technologies 6890N model gas chromatograph equipped with a 5973N mass spectrometric detector (GC-MS; Agilent Technologies, Wilmington, DE). The temperature was programmed from 50°C to 100°C at 10°C/min, then to 200°C at 4°C/min, held for 2 min and finally to 220°C at 4°C/min, held for 12 min. The average helium velocity was 36 cm/s and the split ratio was 100:1. 1 µL of the fatty acid methyl ester was manually injected into the GC-MS and the areas of the total ions from MS with electron ionization for each fatty acid determined the total fatty acid amounts (20).

#### Fluorimetric total choline [free + acetylcholine] assay

Plasma samples were deproteinized using perchloric acid and total choline (free plus acetyl-) was measured using the Amplite Fluorimetric Assay Kit (AAT Bioquest, Inc., Sunnyvale, CA). Plasma was thawed on ice and 250-µL aliquots were transferred into 2-mL Eppendorf tubes. 100 µL 6% perchloric acid was added to each sample, gently mixed, and allowed to sit on ice for 10 min. Samples were then centrifuged at 10,000 × *g* for 5 min, and supernatant transferred to a fresh tube. 50-µL 2M potassium bicarbonate was added to neutralize acid. Samples were then processed according to the kit protocol. Biochemically, this kit hydrolyzes acetylcholine using cholinesterase (21). Choline is then enzymatically oxidized to betaine (22) and the fluorometric reading is characterized based on the reducing equivalents produced. Fluorescence was read using a Bio-Tek Instruments Synergy HT (KC4 software, Winooski, VT).

#### Global DNA methylation patterns

Genomic DNA was isolated from tissues (liver, brain, heart, placenta, and muscle), using proteinase K and phenol/chloroform extraction (23). DNA concentrations were quantified using a NanoDrop Spectrophotometer (Thermo Fisher Scientific, Wilmington, DE). The levels of global DNA 5-methylcytosine (5-mC) were analyzed by MethyFlash methylated DNA Quantification Kit (Colorimetric) according to the manufacturer instructions (Epigenetic Co., Farmingdale, NY). Briefly, the isolated DNA is bound to wells that have a high DNA affinity. The methylated fraction of DNA is detected using capture and detection antibodies and then absorbance is read in a microplate spectrophotometer at 450 nm (24).

### Statistical analysis

The experiment was a 2 × 2 + 1 factorial with an additional positive, full-fed control group, resulting in 5 dietary treatments (Figure 1). Data were analyzed using a complete randomized design using general linear models in SAS (SAS, version 9.3, The SAS Institute, Cary, NC), with gilt as the experimental unit for maternal data and fetus as the experimental unit for fetal data. Differences between dietary treatments for maternal characteristics, descriptive litter characteristics, organ fatty acid content, and plasma total choline concentrations were determined by analysis of variance. When treatment effects for the overall model were found to be significant (*P* < 0.05), a Tukey test was used to determine differences between term fetal weight, term fetal length, term fetal heart girth, percentage litter IUGR, brain, heart, and liver weight of offspring, organ fatty acid content, and plasma total choline concentrations.

DNA methylation data were similarly analyzed by ANOVA/Tukey (as above). In addition, pre-planned contrasts were used to analyze the effects of feed restriction (positive full-feed control compared to pooled restricted diets) on total DNA methylation status in specific tissues and of birth weight on total DNA methylation status by treatment. When assessing differences in LBW (≤900 g) and NBW (>900 g) offspring, data were analyzed according to a 2 × 2 factorial design among restricted dams to detect MV and DHA effects as well as any interaction.

## Results

Body weight gain of malnourished gilts was only 10% of full-fed control dams (*P* < 0.05), but offspring birth weight, length, girth, and percentage of LBW (IUGR) fetuses were not different between treatments (*P* > 0.05; **Tables 3** and **4**). The number of pigs per litter was reduced by 30% in malnourished negative control dams, but there was no detectable difference in total litter weight among the treatments (Table 3). Furthermore, fetal brain weights were reduced by 7% compared to positive controls (*P* < 0.05; Table 4). These reductions were prevented by supplementation of MVs and/or DHA. Additionally, restricted gilts supplemented with only DHA had offspring with lower liver weights (*P* < 0.05; Table 4).

**TABLE 3:** Average change in maternal weight gain, litter size, and litter weight from gilts fed 1 of 5 treatments^1^

Treatments	F MV+D+	R MV–D–	R MV+D–	R MV–D+	R MV+D+	*P* value
Maternal weight gain, kg	49.3 ± 5.97^a^	1.0 ± 5.27^b^	16.0 ± 7.90^b^	–6.1 ± 6.45^b^	7.1 ± 7.07^b^	<0.0001
Piglets/litter	11.4 ± 1.17^a^	7.8 ± 1.07^b^	11.3 ± 1.31^a,b^	10.8 ± 1.17^a,b^	10.3 ± 1.31^a,b^	0.04
Litter weight, kg	13.59 ± 1.27	9.36 ± 1.16	12.31 ± 1.42	11.73 ± 1.27	11.55 ± 1.42	0.21

1 Data represent least square means ± SEs, *n* = 4–6/treatment for maternal data. Means within a row lacking a common superscript letter are different, *P* < 0.05. F MV+D+, positive control, full-fed supplemented with methylating vitamins and DHA; R MV–D–, negative control, restricted basal feed without methylating vitamins or DHA; R MV+D–, restricted basal feed supplemented with methylating vitamins only; R MV–D+, restricted basal feed supplemented with DHA only; R MV+D+, restricted basal feed supplemented with methylating vitamins and DHA.

**TABLE 4:** Average change in average birth weight, length, girth, brain weight, heart weight, and liver weight of fetal piglets and percentage of IUGR of litters delivered by c-section from gilts fed 1 of 5 treatments^1^

	F MV+D+	R MV–D–	R MV+D–	R MV–D+	R MV+D+	*P* value
Birth weight, kg	1.2 ± 0.03	1.1 ± 0.04	1.1 ± 0.04	1.1 ± 0.04	1.1 ± 0.04	0.43
Length, cm	43.8 ± 0.54	42.1 ± 0.58	43.6 ± 0.61	42.7 ± 0.56	43.5 ± 0.64	0.22
Girth, cm	21.9 ± 0.28	22.0 ± 0.31	21.2 ± 0.32	21.5 ± 0.29	22.0 ± 0.34	0.23
Litter IUGR^2^, %	13.3 ± 8.63	15.1 ± 7.89	18.2 ± 9.66	24.4 ± 8.6	15.0 ± 9.66	0.26
Brain weight, g	28.0 ± 0.39^a^	26.1 ± 0.46^b^	28.2 ± 0.44^a^	27.4 ± 0.40^a,b^	28.5 ± 0.45^a^	0.0016
Heart weight, g	8.7 ± 0.23	8.3 ± 0.29	7.9 ± 0.26	8.1 ± 0.24	8.0 ± 0.28	0.17
Liver weight, g	35.2 ± 1.16^a^	35.2 ± 1.43^a^	33.5 ± 1.31^a^	27.4 ± 1.20^b^	33.6 ± 1.37^a^	<0.0001

1 Data represent least square means ± SEs (*n* = 42–58/treatment). Means within a row lacking a common superscript letter are different, *P* < 0.05. F MV+D+, positive control, full-fed supplemented with methylating vitamins and DHA; IUGR, intrauterine growth restriction; R MV–D–, negative control, restricted basal feed without methylating vitamins or DHA; R MV+D–, restricted basal feed supplemented with methylating vitamins only; R MV–D+, restricted basal feed supplemented with DHA only; R MV+D+, restricted basal feed supplemented with methylating vitamins and DHA.

2 IUGR was computed as body weight <900 g.

**FIGURE 2:**
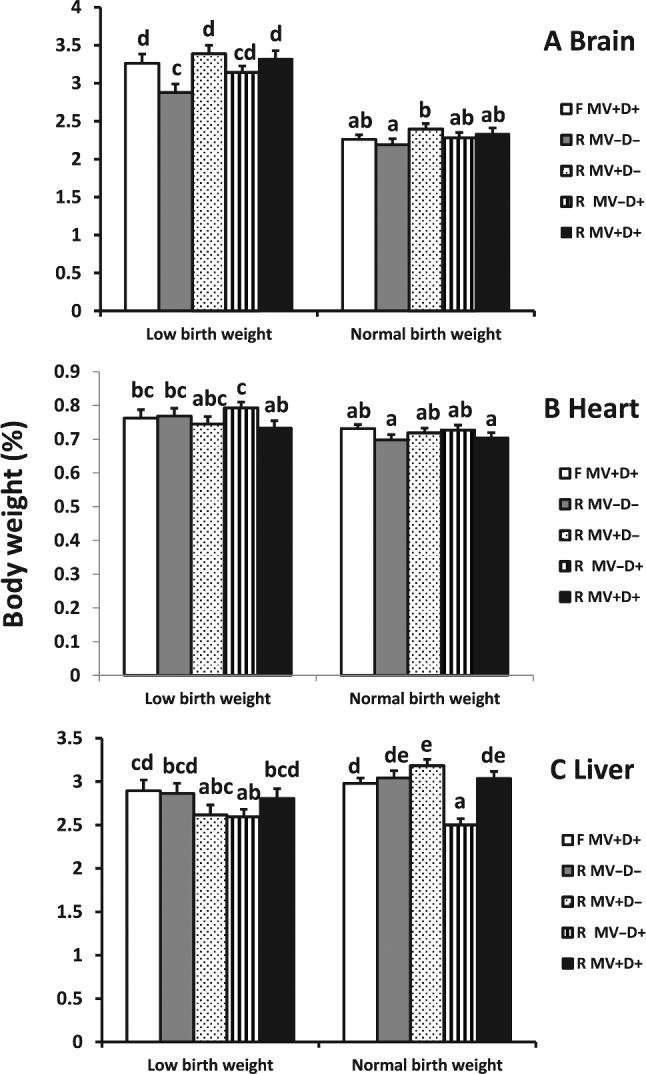
Effect of maternal diet on brain and liver weight (percentage of body weight) of term fetal pigs. Error bars indicate SEM. Bars lacking a common letter are different (*P* < 0.05, *n* = 42–58/treatment). F MV+D+, positive control, full-fed supplemented with methylating vitamins and DHA; R MV–D–, negative control, restricted basal feed without methylating vitamins or DHA; R MV+D–, restricted basal feed supplemented with methylating vitamins only; R MV–D+, restricted basal feed supplemented with DHA only; R MV+D+, restricted basal feed supplemented with methylating vitamins and DHA.

The relative weights of the brain (percentage of body weight) were higher in LBW than NBW fetuses across all the dietary treatments (*P* < 0.0001). The diet with no MV and DHA supplementation reduced the relative brain weight in LBW offspring, but had no impact on NBW offspring from the feed-restricted gilts. Supplemented MVs and/or DHA in the diets of restricted gilts protected from reduction in the brain weight of LBW offspring, and supplemented MVs only increased the relative weight of the brain in NBW offspring (**Figure 2A**). No difference was detected in the relative weight of the heart of NBW piglets between restricted and positive control gilts (*P* = 0.15), although the relative weight of the heart in the LBW fetal pigs from restricted gilts fed a diet with supplementation of DHA was higher than that with supplementation of both choline and DHA (Figure 2B). Supplementation of MVs or DHA in the diet of restricted gilts reduced the relative weight of liver (% of body weight) in the LBW offspring. However, the relative weight of liver in NBW offspring was increased by supplementation of MVs and decreased by supplementation of DHA in the diet of restricted gilts (Figure 2C).

**FIGURE 3:**
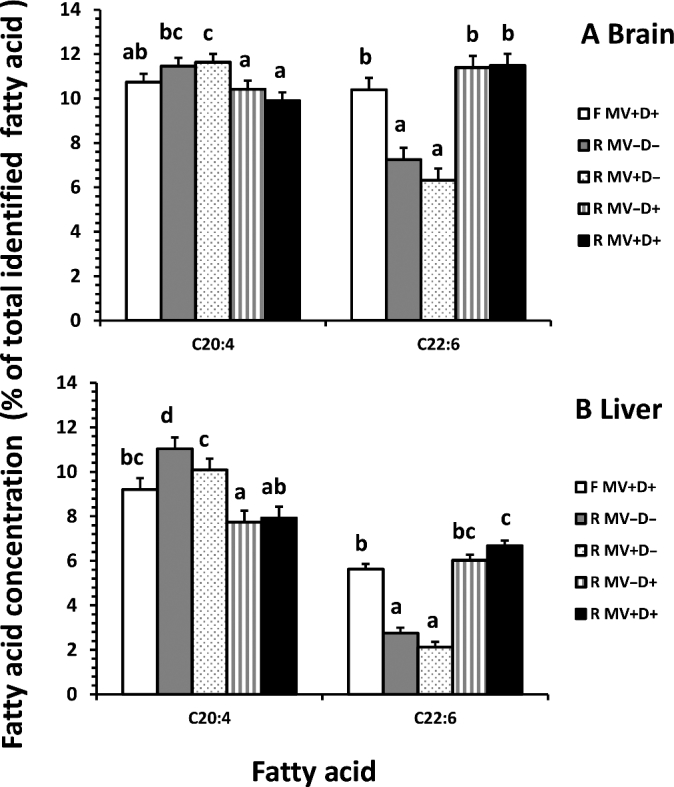
Effect of maternal diet on C20:4n6 (arachidonic acid) and C22:6n3 (DHA) concentrations in the brain and liver tissues of term fetal piglets. Error bars indicate SEM. Bars lacking a common letter are different (*P* < 0.01, *n* = 8/treatment). F MV+D+, positive control, full-fed supplemented with methylating vitamins and DHA; R MV–D–, negative control, restricted basal feed without methylating vitamins or DHA; R MV+D–, restricted basal feed supplemented with methylating vitamins only; R MV–D+, restricted basal feed supplemented with DHA only; R MV+D+, restricted basal feed supplemented with methylating vitamins and DHA.

Brain concentrations of DHA (percentage of total identified fatty acids) were significantly higher in fetuses of dams supplemented with DHA (*P* < 0.01); unsupplemented fetuses showed primary compensation with corresponding increased concentrations of arachidonic acid (ARA; *P* < 0.05; **Figure 3A**). Similar patterns were observed in the liver tissue (*P* < 0.01; Figure 3B).

**FIGURE 4:**
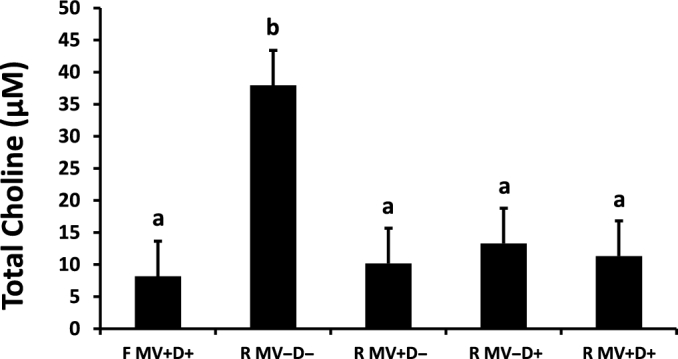
Effect of maternal choline supplementation on plasma total (free + acetyl) choline concentrations (expressed as μM). Error bars indicate SEM. Bars lacking a common letter are different (*P* < 0.01, *n* = 8/treatment). F MV+D+, positive control, full-fed supplemented with methylating vitamins and DHA; R MV–D–, negative control, restricted basal feed without methylating vitamins or DHA; R MV+D–, restricted basal feed supplemented with methylating vitamins only; R MV–D+, restricted basal feed supplemented with DHA only; R MV+D+, restricted basal feed supplemented with methylating vitamins and DHA.

Choline supplementation to maternal diets did not increase fetal plasma total choline concentrations. Rather, choline concentrations were ∼4-fold higher in fetuses from unsupplemented malnourished dams (*P* < 0.001; **Figure 4**).

**FIGURE 5:**
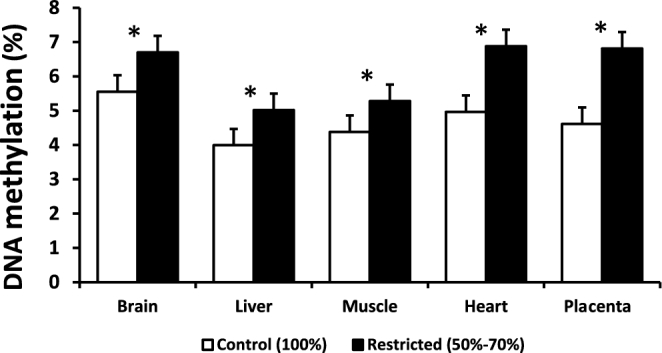
Effect of maternal feed intake on global DNA methylation status in brain, liver, muscle, heart, and placenta of term fetal pigs. Error bars indicate SEM. *Significance *P* < 0.01 (*n* = 8/treatment).

**FIGURE 6:**
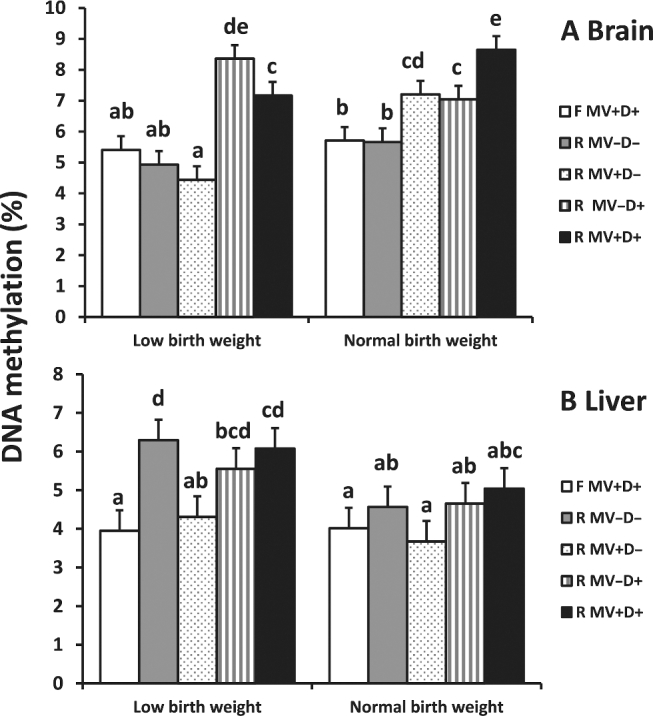
Effect of maternal diet on global DNA methylation status in brain and liver tissues of term fetal pigs. Error bars indicate SEM. Bars lacking a common letter are different (*P* < 0.05, *n* = 8/treatment). F MV+D+, positive control, full-fed supplemented with methylating vitamins and DHA; R MV–D–, negative control, restricted basal feed without methylating vitamins or DHA; R MV+D–, restricted basal feed supplemented with methylating vitamins only; R MV–D+, restricted basal feed supplemented with DHA only; R MV+D+, restricted basal feed supplemented with methylating vitamins and DHA.

Maternal feed restriction significantly increased global DNA methylation status in fetal brain, heart, liver, muscle, and placental tissues (*P* < 0.01; **Figure 5**). Supplementation of DHA in the absence of MVs in this restricted model caused a reversal in global DNA methylation patterns between LBW and NBW offspring in brain tissue (**Figure 6A**). Supplementation with DHA alone or in combination with MVs during a state of global nutrient restriction caused an increase in global DNA methylation in LBW offspring when compared to control and negative control offspring (*P* < 0.05; Figure 6A). Supplementation with any nutrients in this feed-restricted model caused an increase in global DNA methylation in brain tissue of NBW fetuses when compared to control and negative control fetuses (*P* < 0.05; Figure 6A).

Global DNA methylation status did not differ between treatments in liver tissue of NBW fetuses (*P* > 0.05; Figure 6B). Treatment of nutrient-restricted gilts with MVs alone normalized global DNA methylation patterns in liver tissue of LBW offspring (*P* > 0.05). Nutrient-restricted gilts left untreated and supplemented with DHA (R MV–D+), or supplemented with MVs and DHA (R MV+D+), had offspring with increased levels of hepatic DNA methylation when compared to control offspring (*P* < 0.05; Figure 6B).

## Discussion

Nature has favored the success of reproduction, resulting in the ability of the mother to divert nutritional intake to the fetus to ensure survival (25). Here, dams receiving global nutrient restriction gained on average only 10% of the weight that control gilts gained, and the number of piglets from the restricted dams also was reduced by 30% (Table 3). Despite this, the total litter weight did not differ between treatments (Table 3). Although this study was not adequately powered to draw definitive conclusions on reproductive data, it indicates prioritization of nutrients for fetal development. Paired with other published literature (26, 27), this animal model may mimic the ability of women in third-world countries to be able to carry offspring to term despite nutritional inadequacy.

Restriction of nutrients without proper supplementation can cause changes in organ development. Our results showed that global nutrient restriction did stunt growth of the brain (Table 4), and the stunt displayed differences between the LBW and NBW offspring. Maternal global nutrient restriction had a great impact on the relative weight of brain in the LBW but not in the NBW offspring. It was interesting that the impact disappeared in the offspring from gilts fed diets with the supplementation of MVs with choline, demonstrating that the MVs did play an important role in brain development. While impact on functionality was not tested in this experiment, decreased brain size at birth has been shown to impact the amount of gray matter throughout life (28). Additionally, although the importance of omega 3’s in brain development is well established (29) and choline has been implicated in brain development (30), this may indicate a synergistic role for B-vitamins in supporting brain development in utero, as supplementation with a B-vitamin cocktail was able to normalize brain weight to the same extent as DHA supplementation. Additionally, supplementation of DHA in dietary nutrient-restricted gilts increased the relative weight of the heart in LBW offspring and decreased the relative weight of the liver in both LBW and NBW offspring. This could be due to the accumulation of DHA and effect of DHA on the metabolite observed in organs (31), and our data indicated that the impact of maternal diet supplemented with DHA on LBW and NBW offspring could be different.

Clear preferential incorporation of DHA into brain and liver tissues was observed at the expense of ARA incorporation (Figure 3A, B). These results indicate an important role for ω-3 fatty acids, specifically DHA, in fetal brain and liver development. Results seen here also parallel the normalization of brain weight observed in performance data when DHA was supplemented during global nutrient restriction (Table 4).

Lack of dietary choline caused a significant increase in fetal plasma total choline concentration (Figure 4). Despite very low dietary choline, phosphatidylcholine can be produced endogenously through methylation of phosphatidylserine. Phosphatidylcholine can then be converted to free choline, which can then be converted to acetylcholine. Given the importance of choline availability for provision of methyl groups and production of neurotransmitters, fetal concentrations would understandably be protected. In the negative control group, it is possible that conversion of phosphatidylserine to phosphatidylcholine was upregulated and production of free choline was thus able to be maintained to provide fetal access to choline. Specific pathway activity analysis needs to be completed to determine if this is the case.

Overall, feed restriction caused an increase in global DNA methylation patterns in all organs analyzed. We observed an increase in global DNA methylation status in LBW offspring of feed-restricted gilts. Altered global DNA methylation patterns may be indicative of abnormal one-carbon metabolism and lead to long-term health issues (6, 7). However, treatment with MVs during nutrient restriction normalized global DNA methylation patterns in the liver when compared to control fetuses (Figure 6A, B). This adds credence to the idea that appropriate supplementation of key nutrients during global nutrient restriction is a sound corrective nutritional strategy.

Dietary DHA supplementation during nutrient restriction affected performance and organ development, but also played a role in preserving methylation patterns in noncontrol offspring. When DHA was supplemented during nutrient restriction, global DNA methylation patterns were altered in LBW offspring (Figure 6), and liver weight was decreased (Table 4). DHA is a known agonist of fatty acid oxidation (32), and this increase in the absence of an adequate external source of fatty acids may decrease liver weight. Alteration of methylation patterns when DHA is supplemented independently indicates that the role of DHA in epigenetics may be shifted in the presence of choline and other MVs.

Our results clearly indicate maternal preferential diversion of limited exogenous nutrients to fetal development. Normalization of brain weights indicates a role for B-vitamins, choline, and DHA in brain development. In addition, altered hepatic fatty acid composition was observed—elevation of ω-3 fatty acids at the expense of ω-6 fatty acids was evident. In the absence of adequate dietary choline, endogenous production of free choline, possibly from phosphatidylserine, also was apparent. LBW offspring display altered global DNA methylation when compared to NBW counterparts. During nutrient restriction, DHA supplementation altered global DNA methylation patterns in LBW offspring. Treatment with MVs normalized global DNA methylation patterns in liver tissue of LBW offspring.

Our results illustrate the changes in global methylation patterns when comparing LBW and NBW offspring. In support of our hypothesis we observed normalized methylation patterns in liver tissue of LBW offspring when B-vitamins and choline were supplemented. In addition, we have indicated a role for DHA in epigenetic regulation, which adds to current knowledge. Using pigs as an agrimedical model (15), this experiment has laid a foundation for understanding the implications of maternal nutrient restriction and possible nutrition therapy options for supporting fetal growth and development despite nutritional inadequacy. Because swine are a litter-bearing species, there was an advantage in being able to compare LBW to NBW littermates. However, this represents a distinct difference from humans, such that extrapolations should be made carefully.

Future studies are required to quantify the changes on a suborgan level to understand specific metabolic changes that are occurring. Particularly, investigation of suborgan level analysis on brain and liver organs needs to be completed. Our results indicate there may be interesting effects of MVs and DHA on neuronal function and development and hepatic metabolic function. This research also extends application of the agrimedical swine model of IUGR described by Widdowson (33) and Pond et al. (34).

## Notes

Supported in part by the Bill and Melinda Gates Foundation (GCE OPP1061037) and by the North Carolina Agricultural Research Service.

Author disclosures: HKL, XL, SKJ, CM, JS, WF, AB, LG, and JO, no conflicts of interest.

Abbreviations used:

IUGR: intrauterine growth restriction
LBW: low birth weight
MV: methylating vitamin
NBW: normal birth weight
